# Overview of *Candida albicans* and Human Papillomavirus (HPV) Infection Agents and their Biomolecular Mechanisms in Promoting Oral Cancer in Pediatric Patients

**DOI:** 10.1155/2021/7312611

**Published:** 2021-11-02

**Authors:** Lorenzo Lo Muzio, Andrea Ballini, Stefania Cantore, Lucrezia Bottalico, Ioannis Alexandros Charitos, Mariateresa Ambrosino, Riccardo Nocini, Annarita Malcangi, Mario Dioguardi, Angela Pia Cazzolla, Edoardo Brauner, Luigi Santacroce, Michele Di Cosola

**Affiliations:** ^1^Department of Clinical and Experimental Medicine, Università Degli Studi di Foggia, Foggia 71122, Italy; ^2^School of Medicine, University of Bari “Aldo Moro”, Bari 70124, Italy; ^3^Department of Precision Medicine, University of Campania “Luigi Vanvitelli”, Naples 80138, Italy; ^4^Department of Interdisciplinary Medicine, University of Bari “Aldo Moro”, Bari 70124, Italy; ^5^Polypheno Academic Spin Off, University of Bari “Aldo Moro”, Bari 70124, Italy; ^6^Interdepartmental Research Center for Pre-Latin, Latin and Oriental Rights and Culture Studies (CEDICLO), University of Bari “A. Moro”, Bari 70124, Italy; ^7^Department of Emergency and Urgency, National Poisoning Centre, Riuniti University Hospital of Foggia, Foggia 71122, Italy; ^8^Section of Ear Nose and Throat (ENT), Department of Surgical Sciences, Dentistry, Gynecology and Pediatric, University of Verona, Verona 37126, Italy; ^9^Azienda Sanitaria Locale BAT, Trani 76123, Italy; ^10^Department of Oral Sciences, Maxillofacial Surgery Unit, Roma 00161, Italy; ^11^Department of Interdisciplinary Medicine, Microbiology and Virology Unit, School of Medicine, University of Bari “Aldo Moro”, Bari 70124, Italy

## Abstract

Oral carcinoma represents one of the most common malignancies worldwide. Oral squamous cell carcinomas (OSCCs) account over 90% of all oral malignant tumors and are characterized by high mortality in the advanced stages. Early diagnosis is often a challenge for its ambiguous appearance in early stages. Mucosal infection by the human papillomavirus (HPV) is responsible for a growing number of malignancies, particularly cervical cancer and oropharyngeal carcinomas. In addition, *Candida albicans* (*C. albicans*), which is the principal fungi involved in the oral cancer development, may induce carcinogenesis through several mechanisms, mainly promoting inflammation. Medical knowledge and research on adolescent/pediatric patients' management and prevention are in continuous evolution. Besides, microbiota can play an important role in maintaining oral health and therefore all human health. The aim of this review is to evaluate epidemiological and pathophysiological characteristics of the several biochemical pathways involved during HPV and *C. albicans* infections in pediatric dentistry.

## 1. Introduction

Oral carcinoma is the fifth most common malignant tumor worldwide and accounts for the majority of head and neck tumors [1, 2]. During the last decades, there has been a progressive increase in proportion of incidence of oral cancer not related to a known etiologic factor, such as the so-called “oral cancer in young,” a relevant disease in nonsmoker nondrinker (NSND) patients [3]. The topic is matter of long standing debate, and adequate study models to analyze this entity are lacking [4].

Squamous cell carcinomas (OSCCs) are the most common type of oral tumor and can occur in larynx and pharynx too, with over 90% of oral cancers and represents 2-3% of all cancers [5]. Besides, OSCCs are more common in people over the age of 50, while some studies report that 1%-6% occur under the age of 40. There is a special preference for men: in fact, the ratio of men to women in Western societies is 2 : 1 [6]. On the other hand, the incidence of injury in young adults has been noted to be increasing worldwide [4, 7]. In recent years, despite advances in diagnosis and oncologic therapy, the 5-year survival rate of oral carcinoma is still 50–60%, with a slight increase in the United States (US) during the last decade (66%) [6].

The current model of oral and pharyngeal carcinoma development suggests a progressive multistep transition from normal mucosa to OSCCs through a series of progressive histological changes (oral epithelial dysplasia) reflecting the accumulation of genetic and epigenetic abnormalities and genetic susceptibility [5, 6].

The evaluation of the literature and surveillance data concerning oral carcinoma is difficult because this neoplasia often is reported associated with other head and neck malignancies, and report of anatomic subsites is often unclear or can create confusion between its localization between oral cavity and oropharynx.

*Candida albicans* is a commensal fungal species commonly colonizing the human mucosal surfaces [7]. Carriage rates, corresponding to 18.5–40.9% in healthy individuals, are usually higher in individuals with compromised immunity, such as human immunodeficiency virus-positive individuals, diabetes patients, and infants and elder populations [7]. Besides, *C. albicans* can strongly interact with additional oral microorganisms and enforce noteworthy impact on the virulence of polymicrobial biofilms [7]. Furthermore, it is recognized that coinfection of *C. albicans* is strongly associated with increasing diseases in pediatric dentistry, such as severe caries in children (S-ECC) [7].

**Figure 1:**
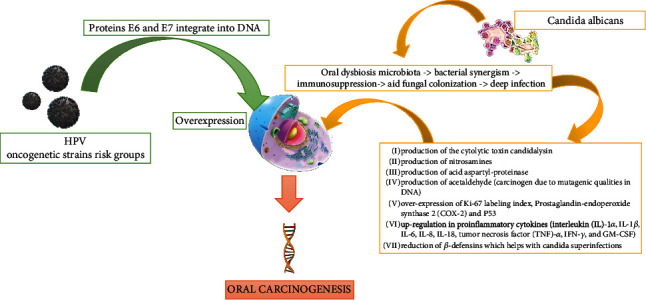
The pathways of carcinogenesis in the oral cavity through the oncogenetic HPV virus strain and *Candida albicans*. Several hypothetical mechanisms have been proposed for the fungal infection that can induce PMODs and malignant lesions in oral epithelium. The dysbiosis of the microbiota can play an important role with the subsequent alteration of the microbiome. There follows a synergism between “bad” bacteria with *Candida albicans* and HPV.

Thus, the purpose of the present paper is to review the current evidence on the role of *C. albicans* and human papillomavirus (HPV), as well their biomolecular mechanisms, in the pathogenesis of potentially malignant oral disorders (PMODs) and oral carcinoma (Figure 1) in pediatric dentistry.

## 2. Risk Factors for Oral Cancer in Pediatric Dentistry

Several risk factors have been involved in the pathogenesis of these lesions, but the mechanisms and the causes of malignant transformations remain unknown. Lifestyle factors, PMODs, modification of the microbiome, systemic sclerosis, genetic disease with dysregulation of DNA metabolism (Zinsser–Engman–Cole syndrome, Fanconi anemia, and Xeroderma pigmentosum), mucosal inflammation and oral mucosa chronic trauma, and hematinic and micronutrient deficiency are the most important causes associated with oral carcinoma [8].

In several Asian countries, the combination of tobacco and/or betel nuts increases the risk for PMODs and oral cancer. In addition, the synergistic effect in consuming large quantities of alcohol and/or tobacco has a high risk associated with consumption and is proportional to the amount of alcohol consumed [9, 10]. Cigarette smoke from combustion releases several carcinogenic chemicals substances such as benzopyrene, dimethylbenzanthracene, nitrosazine, and free radicals [11, 12]. On the other hand, large amounts of alcohol can cause the mitochondrial salivary suppression of aldehyde dehydrogenase ALDH 2 allele gene which leads to high levels of serum acetaldehyde (derived from the ethanol metabolism and turn to acetic acid by ALDH), increasing the risk of carcinogenesis [13, 14].

Virus infections such as HPV (mainly type 16) have been linked to oral carcinomas [15, 16]. Chronic *Candida* spp. infections also appear to be a major risk factor [17, 18]. The risk by HPV strain infections is involved in a growing percentage of oral cancer, but other infectious agents such as the *Candida* spp. (more specifically *C. albicans*) could be involved for their production of endogenous nitrosamines starting from dietary nitrites present in the mouth, particularly in saliva [19, 20].

### 2.1. The Role of Microbiota Dysbiosis in *Candidiasis*

The terms of oral microbiome, oral microbiota, or oral microflora are used for the microorganisms present in the human mouth [21]. The mouth is the second largest and diverse microbiota niche after the gut, harboring over 700 species of bacteria. Every person has his or her own microbiome signature [22]. The human microbiota consists of archaeal cells, bacteria, fungi, viruses, and protozoa. Thus, various bacteria of the oral microbiota have a cross talking with *C. albicans* and HPV by modifying its pathogenicity or the behavior of bacterial population [23].

**Figure 2:**
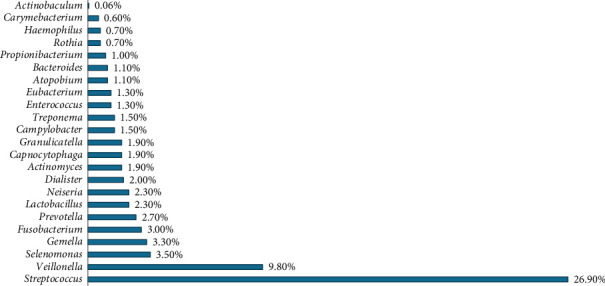
The main relative percentage (%) phyla of oral microbiome reported in eHOMD (source eHOMD, http://www.homd.org/).

The over presenting relative phyla, according to the Human Oral Microbiome Database (eHOMD), as reported in Figure 2, are the *Streptococcus* spp. [24].

**Figure 3:**
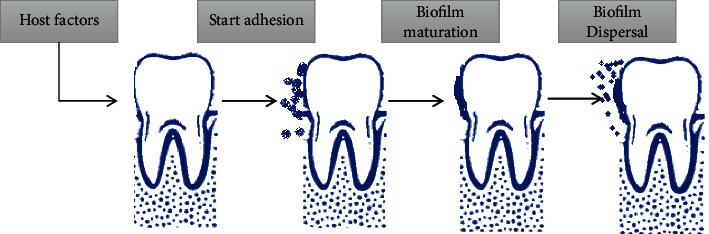
Example stages of biofilm formation process on dental surface (this mechanism occurs throughout the oral cavity). Biofilms are an organized community of microorganisms. Microbes with weak and reversible forces attach first, dental surface. All bacteria that are not immediately removed are rigidly attached to special structures, such as fibrils. Bacteria multiply and offer additional attack sites for other microorganisms. The ability to maintain the consistency of microbes in dental plaque is based on the balance between cooperative and competitive relationships between microorganisms and their host. This microbial homeostasis in combination with host defense prevents the formation of pathogenic microorganism colonization. However, when this community is unbalanced quantitatively and qualitatively, dysbiosis occurs.

The core microbiome is shared to all the persons, while variable microbiome is distinctive for single subject and is due to the lifestyle and to the physiological differences [22]. The mouth has two different types of surfaces on which bacteria can colonize: the hard and the soft tissues, respectively, of the teeth and the oral mucosa [22]. Moreover, the model environment for the development of microorganisms is presented by the oral cavity and related nasopharyngeal regions. In fact, the usual temperature of mouth is about 37°C without significant changes, lead in this way to a stable environment to bacterial survive [23]. In addition, in normal conditions, saliva too has a pH between 6.5 and 7, favorable for most bacteria species [8]. The *Streptococcus mitis* is one of the first bacteria to colonize the oral cavity, establish dental plaque and, under certain conditions (such as the tooth extraction), may cause systemic infection such as endocarditis [25]. *Streptococcus salivarius* electively colonizes the oral mucosa and is over presenting in saliva, acting as an opportunistic pathogen that produce polysaccharides in the presence of fructose, increasing in this way salivary pH [26]. *Streptococcus mutans* (based on the antigens, there are 8 different serotypes), producing bacteriokines and extracellular and intracellular polysaccharides (thus acidifying the oral environment), seems to have a role in the genesis of caries, as well influencing the composition of dental plaque [27]. *Vestibular streptococcus* produces hydrogen peroxide and urease from which ammonia is produced, with a consequent increases of local pH [28]. On the other hand, *Streptococcus anginosus*, usually isolated from the mucous membranes, gingival fissure, and dental plaque, do not secrete polysaccharides playing a protective role in caries development [28]. *Staphylococci* spp. are isolated to a small extent from saliva, gingival cleft of the mucous membranes, and dental plaque (mainly from immunocompromised subjects), with over represented the *S. aureus* [26]. *Lactobacillus species* are members of the oral flora in a small percentage (less than 1%) but increase in tooth areas showing caries, also for the characteristic that it could grow in a low pH environment [23, 27]. Several species of potential opportunistic pathogens, such as *Propionibacterium* and *Corynebacterium*, have been isolated from the oral environment [28]. *Actinomyces* spp. are also common members of dental plaque flora [29]. Other pathogens, such as the *Porphyromonas gingivalis*, *Fusobacterium nucleatum*, and *Treponema denticola*, which are anaerobic bacterium Gram-negative (-), can cause periodontitis and are linked to systemic diseases [30, 31]. Archaeal or archaic cells are only an exceedingly small percentage of the oral microbiome with a limited diversity (very few species and phytotypes) that can be adapted as a small minority of organisms in this environment. *Methanes* have been isolated from the oral cavity and, in fact, in 36% of patients with periodontitis archaeal were detected by in situ fluorescent hybridization [27]. It was found that the archaeal were confined to a subset of human beings and consisted of two different *Rinna fililipi* within the genus *Methanobrevibacter*. The archaeal community in periodontal disease was dominated by a *Methanobrevibacter oralis* type phytologist and a separate subspecies of *Methanobrevibacter* such as *Methanobrevibacter cuticularis*, *Methanobrevibacter filiformi*, *Methanobrevibacter ruminantium*, and *Methanobrevibacter arboriphilius* [21, 22]. Fungi are a small part of the oral microbiome. The predominant species is *Candida albicans.* Other fungal species present in the mouth are *Cladosporium*, *Saccharomycetes*, *Aspergillus* spp. (such as *A. Penicillium*), *Gibberella*, *Cryptococcus*, *Fusarium*, *Rhodotorula*, and *Schizophyllum* [21, 25]. Two species of protozoa were found in physiological flora of the mouth: the *Entamoeba gingivalis* amoeba and the *Trichomonas tenax* [21, 25]. The number of these organisms is high in people with poor oral hygiene and gingivitis and was once considered potential pathogens [28]. At present, saprophytes are considered harmless, and the apparent association with the disease is linked to diet, because a poor oral hygiene can allow an increase in the quantities of food intake and bacterial residues, which are the main nutrients for the protozoa [28]. The conditions of dysbiosis can lead to the imbalance of growth and reduction of some microorganisms present in the oral microbiota. Bacteria are not the only microorganisms present in the periodontal area, and there are also several fungi members. Recent studies in patients with periodontal infections found at least 150 species of fungi belonging to the generate *Candida albicans*, *Ascomycota*, *Basidiomycota*, *Glomeromycota*, and *Chytridiomycota* [29, 30]. This subsequently damages the periodontal tissue, in turn creating nutrients for the bacteria of the dysbiotic microbiome [31]. It has been noted that *Mitis* Group *Streptococci*, *Salivarius* Group *Streptococci*, *S. gordonii*, *S. mutans*, *S. oralis*, *S. sanguinis*, and *S. parasanguini* can interact with *C. albicans*. The *S. mutans* adhering to *C. albicans* interact through three mechanisms. Firstly, *C. ablicans* metabolizes carbohydrates and improves the sugar metabolism of *S. mutans* and *S. mitis*; secondly, *S. mutans* can influence the coding of adhesins in *C. albicans*; finally, the synergism of *S. mutans* and *C. albicans* can promote an increase of growth and invasion of mucous tissues in coinfection [32, 33]. The *S. salivarius* K19 may play a protective role in *C. albicans* infection because it can inhibit adhesion and fungal filamentation. The *S. aureus* may be helped by *C. albicans* in an infection, such as *R. dentocariosa*, and *S. mitis* aids fungal colonization [34, 35]. It has been noted that *Fusobacterium nucleatum*, *S. salivarius*, *A. actinomycetemcomitans*, and *E. faecalis* can have a protective role in a *C. albicans* infections, because it can inhibit the overall virulence and fungal biofilm formation (Figure 3) [36, 37].

Finally, some studies reported several interactions between *C. albicans* and *P. gingivalis* that could be used to increase infectivity [38, 39]. In some patients, the inflammatory reaction in candidiasis can be self-limiting, but in other patients, multiple genetic, epigenetic, or external factors (tobacco, alcohol, diet, diabetes, etc.), as well in synergetic crosstalk with oral bacterial, can cause excessive and chronic inflammation, leading to PMODs and alveolar bone injure [40–42].

Moreover recent evidence points out a causal relationship between specific bacterial infections, such as by *P. gingivalis*, and the development of oral and esophageal carcinoma [43].

### 2.2. Candidiasis Can Generate Oral Precancerous Conditions?

Major predisposing factors to oral candidiasis could be diabetes, mobile prosthesis, antibiotics, antiblastic or inhaled corticosteroid therapy, xerostomia by radiations, or Sjógren's syndrome and HIV infection, presenting clinical forms as angular cheilitis, mucocutaneous (rare), pseudomembranous, hyperplastic, and erythematous (atrophic) [44, 45]. Moreover, current evidence revealed correlation between *Candida* infection and malignant transformation of the oral cavity [46, 47]. In fact, several hypothetical mechanisms have been proposed for *C. albicans interactions with* oral epithelium, leading to PMODs and malignant lesions [48–50]:

- Production of the cytolitic toxin candidalysin
- Production of nitrosamines
- Production of acid aspartyl-proteinase
- Production of acetaldehyde (carcinogen inducing mutations in genes)
- Overexpression of Ki-67 labeling index, prostaglandin-endoperoxide synthase 2 (COX-2), and p53
- Upregulation of proinflammatory cytokines, such as interleukin- (IL-) 1*α*, IL-1*β*, IL-6, IL-8, IL-18, tumor necrosis factor- (TNF-) *α*, interferon gamma (IFN-*γ*), and granulocyte-macrophage colony-stimulating factor (GM-CSF)
- Reduction of *β*-defensins which facilitate Candida superinfections

In a prospective cohort study (between 2007 and 2009) on 103 patients, *Candida* spp. was isolated from 31 (30%) patients with carcinoma and from 33 (32%) patients with PMODs [51]. In another prospective cohort study, it was noted that the presence of *Candida* infections was related to an increased risk of cancer [52]. In addition, some bacteria such as S*. viridans* are able to convert ethanol into acetaldehyde for the presence of the alcohol-dehydrogenase enzyme in them [53–55]. The ability to switch among different phenotypic forms has been thought to contribute to *C. albicans* virulence, and phenotypic switching events in *C. albicans* can be induced by hydroxyurea [55, 56]. Besides, some chemotherapeutic agents, such as 5-fluorouracil, could reduce the susceptibility of *C. albicans* to the antifungal drugs [57]. The genotype “A” of *C. albicans* is more represented in the OSCCs, while the genotype “C” of *C. albicans* is more represented in the leucoplakia [58, 59]. *Candida* leukoplakia (CL) lesions are complex to discriminate from non-*Candida* leukoplakias (NCLs) clinically, but the presence of invading *Candida* hyphae in the superficial layer of epithelium accompanied by infiltratration of polymorphic neutrophils histologically discriminates CL lesions [60, 61]. Moreover, *Candida* was isolated by exfoliative cytology and periodic acid–Schiff (PAS) staining of biopsies in a study that analyzed 44 cases with 59.1% presenting oral leucoplakia, showing *Candida* detection in 62.5% of the cases [62]. Furthermore, in the observed cases with *Candida*, the DNA alterations were higher [62]. In a retrospective study on 136 patients with oral leukoplakia divided into two groups, it was found that lesions had higher degrees of cell abnormalities (epithelial dysplasia) with *Candida* coinfection. The first group presenting multiple oral leucoplakia lesions, *Candida* infection was detected in the 47.9% of the sample (with 28.6% of dysplasia), while in the second group with single oral leucoplakia lesion, *Candida* infection was detected in the 19.0% of the sample (with 20.0% of dysplasia) [63].

### 2.3. The Role of Human Papillomavirus (HPV)

HPVs are members of the Papillomaviridae (PV) family, presenting a circular, with supercoiled and double-stranded DNA, and some are considered as oncogenic promoters [64–66]. The virus has a hexahedral symmetry capsid (consisting of pentameric capsomeres) and has two structural proteins, L1 and L2 [67, 68]. Oral HPV is often sexually transmitted, but nonsexual modes of transmission should be considered, including autoinoculation from skin lesion HPV in adolescent and pediatric patients [69, 70]. The HPV infection is commonly is commomly associated to benign lesions (vulgar warts, warts, focal epithelial hyperplasia, squamous cell papilloma, Bowen's papillomatosis), or to cancerous lesions such as squamous cell carcinoma (SCC) [64]. There must be chafing or small lesions for the virus to enter the epithelium, and direct contact with the skin or mucosa is required for virus transmission. The strain of virus determines the different types of lesion that will be developed, as well as the location of the infection. Hence, HPV can be transmitted in many ways through different abrasions, with sexual intercourse, when the newborn passes through an infected genital tract, and from a variety of self-inoculation positions, for example, by scratching the skin [68].

Since HPV does not have a protein or ribosomal synthesis, for its proliferation, it employs the genetic mechanism of the host cell [69, 70]. The virus uses, to direct the metabolic functions of the host cell in its favor, the production of viral messenger RNA, which is produced by transcription of viral genetic material. The genome is divided into three parts. The “E” (early region) which codes for proteins necessary for the viral DNA genome duplication. It includes 7-9 open reading frames (ORFs), coding regions for proteins E1, E2, E4, E5, E6, E7, and E8 (although E5 and E8 ORF are not present in the genomes of all HPV types). The “L” (late region) which encodes the structural proteins of the HPV capsid virus (L1 and L2). Finally, the URR or NCR (upstream regulatory region or noncoding region) comprised between “E” and “L” regions and regulates the function of the viral genome, in addition to four binding sites to E2 proteins and multiple binding sites of the transcription [69–71].

The differences between different strains of the virus are evident when comparing lesions of the same epithelial area. In fact, cell proliferation obtained from the expression of E6 and E7 proteins, due to infection with oncogenic HPV virus strains, facilitates expansion of lesion, with a higher risk of metastasis [71, 72].

### 2.4. Which Are the Pathways of Oncogenesis in HPV Infection?

The receptor that HPV uses to enter cells is integrin A6 [71]. On one hand, there are many types of HPV such as 16, 18, 31, and 52, and on the other hand, they are characterized by a high potential for malignancy and also have a high risk of metastasis (Table 1) [73, 74].

The role of the virus on carcinogenesis is due to proteins E6 and E7 that integrate into host DNA. These two proteins could induce the neoplastic growth, since they block the two tumor suppressor proteins, the retinoblastoma protein (pRb) and p53, which regulate the transition from the G1 phase of the cell cycle to S. E7 binds to p53 blocking its ability to interact with the transcription factor E2F [75, 76]. Therefore, cells characterized by E7 overexpression lose the control of the transition from G1 to S phase, which causes continuous cell cycles and therefore cell proliferation without suppression, as well, degradation of the pRb [76].

**Figure 4:**
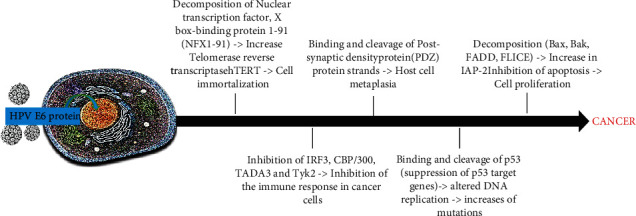
Integration of HPV DNA into the host genome leading to the E6 protein overexpression and to carcinogenic processes.

Levels of p53 in normal cells are exceptionally low. During the E7 overexpression, an increase in p53 was shown for the inhibition of its breakdown in normal cells which is in turn regulated by MDM2 (mediator of DNA damage 2) [77, 78]. E7 also interacts with many other factors; among these, the CDK (cyclin-dependent kinase) inhibits p27 and p21, which induce the dysregulation of cell cycle [78]. E7 forms protein complexes with pRb, p107, and p130 and participates in E6 cleavage of p53 via the E6 complex, E6AP (associated protein E6), and p53 [79, 80]. Dysregulation of pRb phosphorylation-dephosphorylation is an early event. There is a gradual reduction of pRb expression in dysplastic lesions and in neoplastic process [81]. Briefly, the Rb protein pathway could induce carcinogenesis by inactivating cell cycle regulators [82]. Activation of p53 turns on the production of the CD-kinase inhibitor protein, p21WAF1 which contributes to cell cycle regulation by acting on different CD-kinase/cyclin complexes [83]. Thus, failure of p53 has been observed to cause carcinogenesis. This is attributed to the mutation of the genetic locus 17p13. Furthermore, E6 sequentially causes the cleavage of p53 [84]. Oncoprotein E7 causes p16 overexpression. In fact, the gene that codes for the regulatory protein p16 is found in chromosome 9p21, and its mutation is correlated with OSCCs [85, 86] (Figure 4).

**Figure 5:**
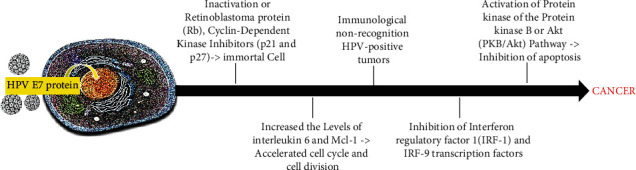
Integration of HPV DNA into the host genome leading to E7 overexpression and to carcinogenic processes.

Besides, E6 inhibits the activity of p73, a type 16 homologous p53. HPV blocks cell apoptosis by inhibiting Bax gene expression in keratinocytes. This results in increased mutations in the DNA of the cells. The apoptosis can be inhibited by E7 binding to the tumor necrosis factor 1 receptor (TNF-R1) [87], originating a variety of bimolecular effects that promote carcinogenesis (Figure 5).

According to carcinogenesis model proposed by Califano et al., healthy tissues near the lesions show the same pattern of mutations (loss of heterozygosity) of neoplastic cells, since all population come from a single mutant progenitor cell [88]. Another model reports that the progenitor cell of the basal layer acquires a genetic mutation, which it transfers to its daughter cells. The result is the creation of a mass of cells that grows, strains, and also affects nearby tissues, in the development of lesions (clinically, they appear as leukoplakia or erythroplakia). In the final stage, specific clone cells after further mutations acquire a tumoral genotype. In this context, loss of heterozygosity at loci 9p21 and 3p21 appears to increase the risk of malignant recurrence [89, 90]. A mutation in the type of gene duplication has been shown to be involved in oral carcinogenesis [91].

Replication of genetic material in the TERT (telomerase reverse transcriptase) protein coding gene results in overexpression of the TERT human protein (hTERT). Studies conducted on OSCCs have shown an increase in both telomerase activity and hTERT expression [92, 93]. This hTERT expression occurs not only in cancerous cells but also around in the normal epithelium in the early course of carcinogenesis [94]. The spindle assembly checkpoint (SAC) describes the way point to control mitosis, and Cdc20 is a SAC protein able to activate the anaphase promoting complex (APC) which is a key pathway factor and is also responsible for the formation of aneuploidy cells [95–97].

It has been noted that in 70% of oral tumors there is an overexpression of the Cdc20 mRNA [98, 99]. Besides, as further bimolecular mechanisms in the etiology of oral cancer, several studies reported the role of the epidermal growth factor (EGF), the mitogen-activated protein kinase (MAPK) pathway, the PI3K/AKT/mTOR pathway, the signal transducer and activator of transcription (STAT) pathway, TGF (transforming growth factor), NF-*κ*B (nuclear factor *κ*B), and Wnt/*β*-catenin [100–102]. The ERbB1 gene, responsible for EGF encoding, is found on chromosome 7p12 and has been associated with increased activity in oral cancer [103, 104]. ERbB1 dysregulation in head and neck squamous epithelial neoplasms is attributed more to its overexpression than to the presence of mutations [105, 106]. TGF promotes tumor progression by increasing angiogenesis and reducing sensitivity to the immune system. This action is caused by the tolerance of tumor cells to TGF-induced apoptosis [107, 108]. The TGF*β*R-II mutation is important, and thus, a reduction of the TGF*β*R-II/TGF*β*R-I ratio (suspension/development) is determined, and thus, the protective role of TGF is canceled [109]. A gradual decrease in expression of TGF-*β*, TGF*β*R-I, and TGF*β*R-II was observed in the different stages of carcinogenesis. In cancer cells, there is a reduction in TGF*β*R-II levels, and this can promote the development and growth of oral cancer, acting as an indicator of differentiation and therefore of aggressive behavior [110]. Activation of the Wnt/*β*-catenin pathway induces *β*-catenin release and subsequently cytoplasmic aggregation and its transport to the nucleus, through the interaction with certain genes (with inhibition of apoptosis and increase of cell proliferation) such as COX-2, cyclin D1, and cMyc [105, 111]. Furthermore, this pathway increases the expression of metalloproteinases, which catalyze the basement membrane and the dense structure of the epithelium, favoring infiltration [111]. Lastly, HPV proteins E6 and E7 can regulate the epigenetic mechanisms, as DNA methylation, histone modification, chromatin remodeling, and miRNA production in cell host or viral genes. Therefore, on one hand, E6/E7 induced DNA methylation shut out normal epigenetic processes, and on the other hand, E7 binds and modulates methyltransferase activity [112, 113].

## 3. Conclusions

Nowadays, several suggestive and consistent correlations between *C. albicans* and HPV infection in oral cancer progression in adolescent/pediatric patients have been reported worldwide. *Candida* spp. and in particular *C. albicans* can produce carcinogens such as nitrosamine or promote the development of oral carcinoma. HPV are involved in the pathogenesis of different types of cancer. In particular, from literature data, it emerges that HPV infection plays an important role in the risk of PMODs of the oral mucosa and consequently in the dysplastic and malignant transformation of these lesions. Based on existing evidence, we can also conclude that, in the composition of the microbiome associated with OSCCs, there are no specific species to implicate in its etiology, of course excluding oncoviruses (i.e., HPV) or fungi (i.e., *C. albicans*) that are associated with oral cancer. Large multicenter trials are required in order to study the biological behavior and formulate treatment strategies in the management of the same. Perhaps, these findings will produce interest in the possible association among oral microbiota dysbiosis, *C. albicans*, HPV, and oral cancer and spur controlled prospective clinical studies in this field.

## Figures and Tables

**Table 1:** There are over 450 types of HPV. The HPV's genotypes are divided into 4 groups according to the associate oncogenic risk by IARC/WHO (source from https://monographs.iarc.who.int/agents-classified-by-the-iarc/).

Group	HPV virus
1	Carcinogenic to humans: human papillomavirus types 16, 18, 31, 33, 35, 39, 45, 51, 52, 56, 58, and 59 (the HPV types that have been classified as carcinogenic to humans can differ by an order of magnitude in risk for cervical cancer)
2A	Probably carcinogenic to humans: human papillomavirus type 68
2B	Possibly carcinogenic to humans: human papillomavirus types 26, 53, 66, 67, 70, 73, and 82; human papillomavirus types 30, 34, 69, 85, and 97 (classified by phylogenetic analogy to the HPV genus alpha types classified in group 1); human papillomavirus types 5 and 8 (in patients with epidermodysplasia verruciformis)
3	Not classifiable as to its carcinogenicity to humans: human papillomavirus genus beta (except types 5 and 8) and genus gamma; human papillomavirus types 6 and 11

## Data Availability

The data used to support the findings of this study are included within the article.
